# Neurologic outcome of VZV encephalitis one year after ICU admission: a multicenter cohort study

**DOI:** 10.1186/s13613-022-01002-y

**Published:** 2022-04-05

**Authors:** Adrien Mirouse, Romain Sonneville, Keyvan Razazi, Sybille Merceron, Laurent Argaud, Naïke Bigé, Stanislas Faguer, Pierre Perez, Guillaume Géri, Claude Guérin, Anne-Sophie Moreau, Laurent Papazian, René Robert, François Barbier, Frédérique Ganster, Julien Mayaux, Elie Azoulay, Emmanuel Canet

**Affiliations:** 1 Service de Médecine Intensive et Réanimation, Hôpital Saint-Louis, AP-HP, Paris, France; 2 Université de Paris, Paris, France; 3 Service de Médecine Intensive et Réanimation, Hôpital Bichat, APHP, Paris, France; 4 Service de Médecine Intensive et Réanimation, Hôpital Henri Mondor, Créteil, France; 5 Service de Réanimation Polyvalente, Hôpital André Mignot, Le Chesnay, France; 6 Service de Médecine Intensive et Réanimation, Hôpital E. Herriot, Hospices Civils de Lyon, Lyon, France; 7 Service de Médecine Intensive et Réanimation, Hôpital Saint-Antoine, APHP, Paris, France; 8 Département de Néphrologie et Transplantation d’organes – Unité de Réanimation, CHU de Toulouse, Toulouse, France; 9 Service de Réanimation Médicale, Hôpital Brabois, Nancy, France; 10 Service de Médecine Intensive et Réanimation, Hôpital Cochin, APHP, Paris, France; 11 Service de médecine intensive et réanimation, Groupement Hospitalier Nord, Hospices Civils de Lyon, Université de Lyon, INSERM 955, Créteil, France; 12 Service de Médecine Intensive et Réanimation Groupement Hospitalier Centre, Hôpital Edouard Herriot, Lyon, France; 13 Service de Réanimation Polyvalente, CHRU de Lille - Hôpital Roger Salengro, Lille, France; 14 Service de Réanimation des Détresses Respiratoires et Infections Sévères, Hôpital Nord, AP-HM, Marseille, France; 15 Service de Réanimation Médicale, CHU de Poitiers, Poitiers, France; 16 Service de Réanimation Médicale, Hôpital la Source, Orléans, France; 17 Service de Réanimation Médicale, Hôpital E. Muller, Mulhouse, France; 18 Service de Médecine Intensive et Réanimation, Hôpital Pitié-Salpêtrière, AP-HP, Paris, France; 19 Service de Médecine Intensive et Réanimation, CHU de Nantes, Nantes, France; 20 Département de Médecine Interne et Immunologie Clinique, Hôpital Pitié-Salpêtrière, APHP, 83 boulevard de l’hôpital, 75013 Paris, France

**Keywords:** Varicella-zoster virus, Encephalitis, Virus, Intensive care unit, Long-term prognosis, Acute brain injury

## Abstract

**Background:**

Varicella-zoster virus (VZV) is one of the main viruses responsible of acute encephalitis. However, data on the prognosis and neurologic outcome of critically ill patients with VZV encephalitis are limited. We aimed to describe the clinical features of VZV encephalitis in the ICU and to identify factors associated with a favorable neurologic outcome. We performed a multicenter cohort study of patients with VZV encephalitis admitted in 18 ICUs in France between 2000 and 2017. Factors associated with a favorable neurologic outcome, defined by a modified Rankin Score (mRS) of 0–2 1 year after ICU admission, were identified by multivariable regression analysis.

**Results:**

Fifty-five patients (29 (53%) men, median age 53 (interquartile range 36–66)) were included, of whom 43 (78%) were immunocompromised. ICU admission occurred 1 (0–3) day after the onset of neurological symptoms. Median Glasgow Coma Score at ICU admission was 12 (7–14). Cerebrospinal fluid examination displayed a median leukocyte count of 68 (13–129)/mm^3^, and a median protein level of 1.37 (0.77–3.67) g/L. CT scan and MRI revealed brain lesions in 30% and 66% of the cases, respectively. Invasive mechanical ventilation was implemented in 46 (84%) patients for a median duration of 13 (3–30) days. Fourteen (25%) patients died in the ICU. One year after ICU admission, 20 (36%) patients had a favorable neurologic outcome (mRS 0–2), 12 (22%) had significant disability (mRS 3–5), and 18 (33%) were deceased (lost to follow-up *n* = 5, 9%). On multivariable analysis, age (OR 0.92 per year, (0.88–0.97), *p* = 0.01), and invasive mechanical ventilation (OR 0.09 CI 95% (0.01–0.84), *p* = 0.03) reduced the likelihood of favorable neurologic outcome.

**Conclusion:**

One in every three critically ill patients with VZV encephalitis had a favorable neurologic outcome 1 year after ICU admission. Older age and invasive mechanical ventilation were associated with a higher risk of disability and death.

**Supplementary Information:**

The online version contains supplementary material available at 10.1186/s13613-022-01002-y.

## Background

Acute encephalitis is a neurologic syndrome characterized by the inflammation of the brain parenchyma from a wide range of infectious and autoimmune causes. This severe condition is associated with mortality rates up to 15%, but also with significant morbidity among survivors with a high rate of disability and neuropsychiatric sequelae [1]. The widespread of conjugate vaccines has led to a reduction in the incidence of bacterial meningitis with viruses now being the most common cause of central nervous system (CNS) infections [2]. Moreover, the routine use of polymerase chain reaction (PCR) assays has greatly increased our ability to identify in the cerebrospinal fluid (CSF) many neurotropic viruses known to cause infectious encephalitis [3]. About 40% of patients with encephalitis require admission to the intensive care unit (ICU), and one-fourth invasive mechanical ventilation, for close monitoring or management of coma, seizures or other complications (aspiration pneumonia, autonomic instability or cardiac arrhythmia) [4]. The outcome of acute viral encephalitis in the ICU is estimated to be poor, although data are limited to specific etiologies (herpes simplex virus (HSV)-1 or -2) or small single-center experiences [5–8].

Varicella-zoster virus (VZV) is one of the eight herpes viruses known to cause human infections and is the second cause of acute encephalitis in France after herpes simplex virus (HSV) 1 and 2 [2, 3, 9]. Its incidence has grown over the last decade [10]. The most common neurologic symptoms of VZV infection are meningoencephalitis, cerebellitis, stroke, myelopathy and retinitis.

Therefore, we conducted a large multicenter cohort study to report the clinical features, ICU management, and outcome of VZV encephalitis in ICU patients. We aimed to investigate the association between the use of invasive mechanical ventilation and the neurologic outcome 1 year after ICU discharge. We hypothesized that the use of invasive mechanical ventilation in patients with VZV encephalitis would negatively impact neurologic outcome.

## Methods

This study was approved by the French Intensive Care Society Ethics Committee (#15-12) on March the 16th 2015, and received an authorization to use patients’ data from the French Data Protection Agency (no. 1868870). According to French law, a waiver of informed consent was obtained.

### Study design, setting, and population

We conducted a multicenter retrospective cohort study in 18 French ICUs (see Additional file 1: Table S1). We identified all adults (≥ 18 years of age) admitted to the participating ICUs between January 1, 2000, and January 1, 2017, and registered in the electronic hospital databases with any of the codes for VZV encephalitis by using the International Classification of Diseases 10th Revision (ICD-10) coding system (B01.1, B01.0, B02.0, B02.1). All patients’ medical records were reviewed by 2 investigators (AM and EC) to confirm the diagnosis of VZV encephalitis. The diagnosis of encephalitis was confirmed if patients had an acute change in mental status or behavior ≥ 24 h mentioned in their medical charts, with at least 2 of the following manifestations: fever within 72 h before or after presentation, generalized or partial seizures, new onset of focal neurological findings, lumbar puncture with CSF white blood cell count ≥ 5/mm^3^, neuroimaging or electroencephalogram (EEG) abnormalities suggestive of encephalitis [11]. A positive PCR for VZV DNA in a CSF sample was mandatory for the diagnosis of VZV encephalitis.

### Data collection

For each patient, data were extracted manually from the medical chart. We obtained data for baseline patient characteristics, including demographics, comorbidities, chronic medications, and past medical history. Clinical and laboratory data, neuroimaging, and EEG were extracted from the medical charts. Immunosuppression was defined as chronic use of steroids (> 3 months) or other immunosuppressive drugs, solid organ transplantation, HIV infection, hematologic malignancy or solid tumor treated by chemotherapy during the past 5 years. Neurologic symptoms were classified as confusion, coma, seizures, status epilepticus, motor or sensitive impairment, cerebellar syndrome, and cranial nerves disorders. Mental status at baseline was graded using the Glasgow Coma Scale (GCS). Coma was defined as a GCS < 8. Other VZV-related manifestations were extracted from the medical chart, including chicken pox skin rash, hepatitis, pneumonia, and digestive symptoms. Disease severity was assessed using the Sequential Organ Failure Assessment (SOFA) at day 1 after ICU admission [12]. Acute kidney injury (AKI) was defined according to the Kidney Disease Improving Global Outcomes criteria [13]. High-dose steroids were defined as more than 30 mg/day of equivalent prednisone.

### Outcome

Two physicians (AM, EC) assessed neurologic outcomes by reviewing the medical charts and or by contacting the general practitioner in charge of the patient and or the patient. The outcome was assessed at ICU discharge, 6 months, and 1 year after ICU discharge using the modified Rankin Scale (mRS) (scores range from 0 to 6, with higher scores indicating greater disability and score 6 indicating death) [14]. For this study, a favorable neurologic outcome was defined as an mRS score of 0 (no symptoms at all), 1 (no significant disability) or 2 (slight disability).

### Objectives

The primary objective of the study was to identify factors associated with a favorable neurologic outcome 1 year after ICU discharge. The secondary objectives were to describe the clinical features, treatments, and outcomes of critically ill patients with VZV encephalitis.

### Statistical analysis

Continuous variables are described as median and interquartile range (IQR) and compared using Wilcoxon’s test; categorical variables are shown as counts (percent) and compared using exact Fisher test. We did not use any technique to replace missing data; missing data are reported. The occurrence of favorable 1-year neurologic outcome (versus unfavorable 1-year neurologic outcome) was analyzed as a binary variable. Logistic regression analyses were performed to identify variables which were associated with the occurrence of favorable 1-year neurologic outcome, with estimated odds ratios (OR) and 95% confidence intervals (95% CI). The multivariable model selected to identify factors independently associated with favorable 1-year neurologic outcome was also a logistic regression model. For the multivariable model, we preselected candidate variables which plausibly fit with complicated hospital stay based on knowledge from the literature and our assumptions (age, invasive mechanical ventilation and underlying immunosuppression). We carefully checked to avoid collinearity between variables and we applied the rule of selecting approximately 1 variable per 7 events. For the number of events of the smallest group (*n* = 20), a maximum of 3 variables were included. All tests were two-sided, and p-values lower than 5% were considered to indicate significant associations. Statistical tests were conducted using the R statistics software, version 3.5.0 (R Foundation for Statistical Computing, Vienna, Austria; www.R-project.org/).

## Results

### Study population

**Fig. 1:**
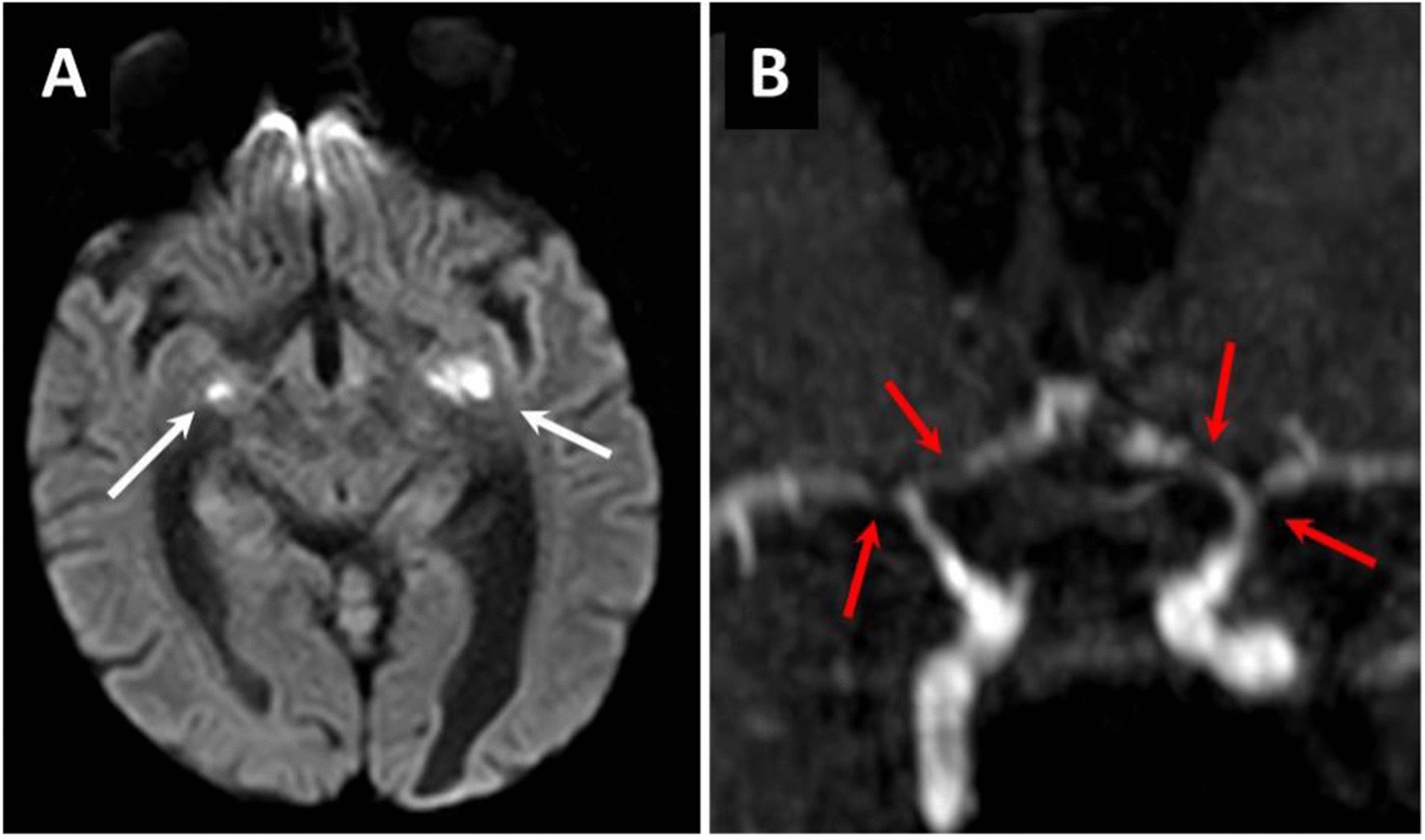
Imaging characteristics of VZV encephalitis and vasculitis from brain MRI. **A** 18-year-old man was admitted to the ICU for fever, headaches and altered consciousness. He underwent a second kidney transplantation 2 years ago. The onset of neurologic symptoms started 2 days before ICU admission and invasive mechanical ventilation was implemented at day 2 for coma. The lumbar puncture revealed acute lymphocytic meningitis and the PCR for VZV was positive. Brain MRI showed multiple recent ischemic lesions of the brain parenchyma (**A**: white arrows) and vasculitis with multiple stenosis of the circle of Willis arteries (**B**: red arrows). He was treated with intravenous acyclovir 10 mg/kg/8 h and steroids (methylprednisolone 1 mg/kg). He recovered gradually and was extubated after 4 days of invasive mechanical ventilation. The patient was ultimately discharged alive 8 days after ICU admission

During the study period, 55 critically ill patients were coded as having VZV encephalitis. A detailed analysis of the medical files confirmed the diagnosis for the 55 patients. Table 1 reports their main features. Median age was 53 (36–66) years and 29 (53%) were men. Forty-three (78%) patients were immunocompromised with chronic use of steroids, malignancy and solid organ transplantation being the most common causes of immunosuppression. Patients were admitted in the ICU 1 (0–3) day after the onset of neurologic symptoms. The main reasons for ICU referral were coma (*n* = 22, 40%) and confusion (*n* = 20, 36%). At ICU admission, the median GCS was 12 (7–14) and a body temperature higher or equal to 38.3 °C was noted in 34 (62%) patients. Two-thirds of the patients had a typical chickenpox skin rash associated with the neurologic symptoms. All patients had abnormal CSF examination with a median leukocyte count of 68 (13–129)/mm^3^ with predominance of lymphocytes (70% [61%–82%]), and a median protein level of 1.37 (0.77–3.67) g/L. A brain imaging was performed in 42 (76%) patients, including 33 (66%) computed-tomography (CT) scan and 30 (55%) magnetic resonance imaging (MRI), abnormal in 10 (30%) and 21 (70%) cases, respectively. Twelve (22%) patients had a normal CT-scan followed by an abnormal brain MRI. Brain MRI analysis displayed brain parenchyma hyperintensities consistent with encephalitis in 10 (33%) patients, cerebral vasculitis in 8 (27%) patients (ischemic lesions in 5 patients, microbleeds in 2 patients and cerebral hemorrhage in one patient), and brain edema in 3 (10%) patients (Fig. 1). Brain MRI lesions involved frontal lobes in 6 (20%) patients, temporal lobes in 4 (13%) patients, parietal lobes in 4 (13%) patients, occipital lobes in 3 (10%) patients, basal ganglia in 3 (10%) patients, brainstem in 3 (10%) patients, and cerebellum in 3 (10%) patients. EEG abnormalities were noted in 18/24 (75%) patients. Main abnormalities included seizures or post-ictal changes (*n* = 5, 21%), and non-specific encephalopathy (*n* = 13, 54%).

**Table 1:** Baseline characteristics of the 55 study participants

Variable	Patients *n* = 55
Age, years, median [IQR]	53 [36–66]
Male sex, *n* (%)	29 (53%)
Underlying immunosuppression, *n* (%)	43 (78%)
Steroids	20 (36%)
Solid organ transplant	12 (22%)
HIV	11 (20%)
Hematological malignancy	11 (20%)
Solid neoplasm	3 (5%)
Other causes of immunosuppression^a^	8 (15%)
Reason for ICU admission, *n* (%)	
Confusion	22 (40%)
Coma	20 (36%)
Status epilepticus	10 (18%)
Neurologic deficits	3 (5%)
VZV-related organ involvement, *n* (%)	
Skin rash	37 (67%)
Pneumonia	11 (20%)
Hepatitis	11 (20%)
Neurological symptoms	55 (100%)
Confusion	27 (49%)
Coma	20 (36%)
Seizures	18 (33%)
Headaches	18 (33%)
Neurologic deficits	17 (31%)
Ataxia	3 (5%)
Time from the onset of neurological symptoms to ICU admission, days, median [IQR]	1 [0–3]
Clinical parameters at ICU admission, median [IQR]	
Temperature	38.5 [37.3–39.3]
SOFA	6 [4–9]
GCS	12 [7–14]
Cerebrospinal fluid examination, median [IQR]	55 (100%)
Leukocytes, /mm^3^	68 [13–129]
Lymphocytes, /mm^3^	70 [27–95]
Neutrophils, /mm^3^	25 [3–40]
Protein, g/L	1.37 [0.77–3.67]
Glucose, mmol/L	3.8 [2.6–5.0]
Positive VZV PCR, *n* (%)	55 (100%)
Brain CT-scan, *n* (%)	33 (60%)
Abnormal findings	10 (30%)
Brain MRI, *n* (%)	30 (55%)
Abnormal findings	21 (70%)
EEG, *n* (%)	24 (44%)
Abnormal findings	18 (75%)

^a^Other causes of immunosuppression: connective tissue disorder treated with immunosuppressive drugs (*n* = 4), common variable immunodeficiency (*n* = 2), pregnancy (*n* = 2), cirrhosis (*n* = 2)

CT, computed tomography; EEG, electroencephalogram; GCS, Glasgow Coma Scale; HIV, human immunodeficiency virus; ICU, intensive care unit; IQR, interquartile; MRI, magnetic resonance imaging; SOFA, Sequential Organ Failure Assessment; PCR, polymerase in chain reaction; VZV, varicella-zoster virus

### ICU management and outcomes

Table 2 provides details about the treatments used in the ICU. Two-thirds of the patients were treated with 10 mg/kg/8 h of intravenous acyclovir and one-third with 15 mg/kg/8 h for a median duration of 14 (9–19) days. Eight (15%) patients received an additional antiviral drug (foscavir, *n* = 6, ganciclovir, *n* = 2) and 3 (5%) were treated with intravenous immunoglobulins. High-dose steroid pulses were administered to 4 (7%) patients for the treatment of VZV-related brain vasculitis (Fig. 1). During the ICU stay, 18 (33%) patients had seizures and 12 (22%) developed status epilepticus. Mechanical ventilation was implemented in 46 (84%) patients for a median duration of 13 (3–30) days. Intubation occurred on day 1 after ICU admission in 43 (78%) patients. AKI was diagnosed in 28 (51%) patients and 15 (27%) required renal replacement therapy.

**Table 2:** ICU management and outcome

Variables	Patients
Neurocritical care, *n* (%)	
Acyclovir	55 (100%)
High dose of steroids	4 (7%)
Anticonvulsant therapy	12 (22%)
Life-sustaining therapies	
Invasive mechanical ventilation (IMV), *n* (%)	46 (84%)
Duration of IMV, days, median [IQR]	13 [3–0]
Vasopressors, *n* (%)	22 (40%)
Renal replacement therapy, *n* (%)	15 (27%)
Outcome	
ICU mortality, *n* (%)	14 (25%)
ICU length of stay, days, median [IQR]	15 [6–38]
Hospital length of stay, days, median [IQR]	35 [17–55]
One-year mortality	18 (33%)

AKI, acute kidney injury; ICU, intensive care unit; IQR, interquartile; VZV, varicella-zoster virus

**Fig. 2:**
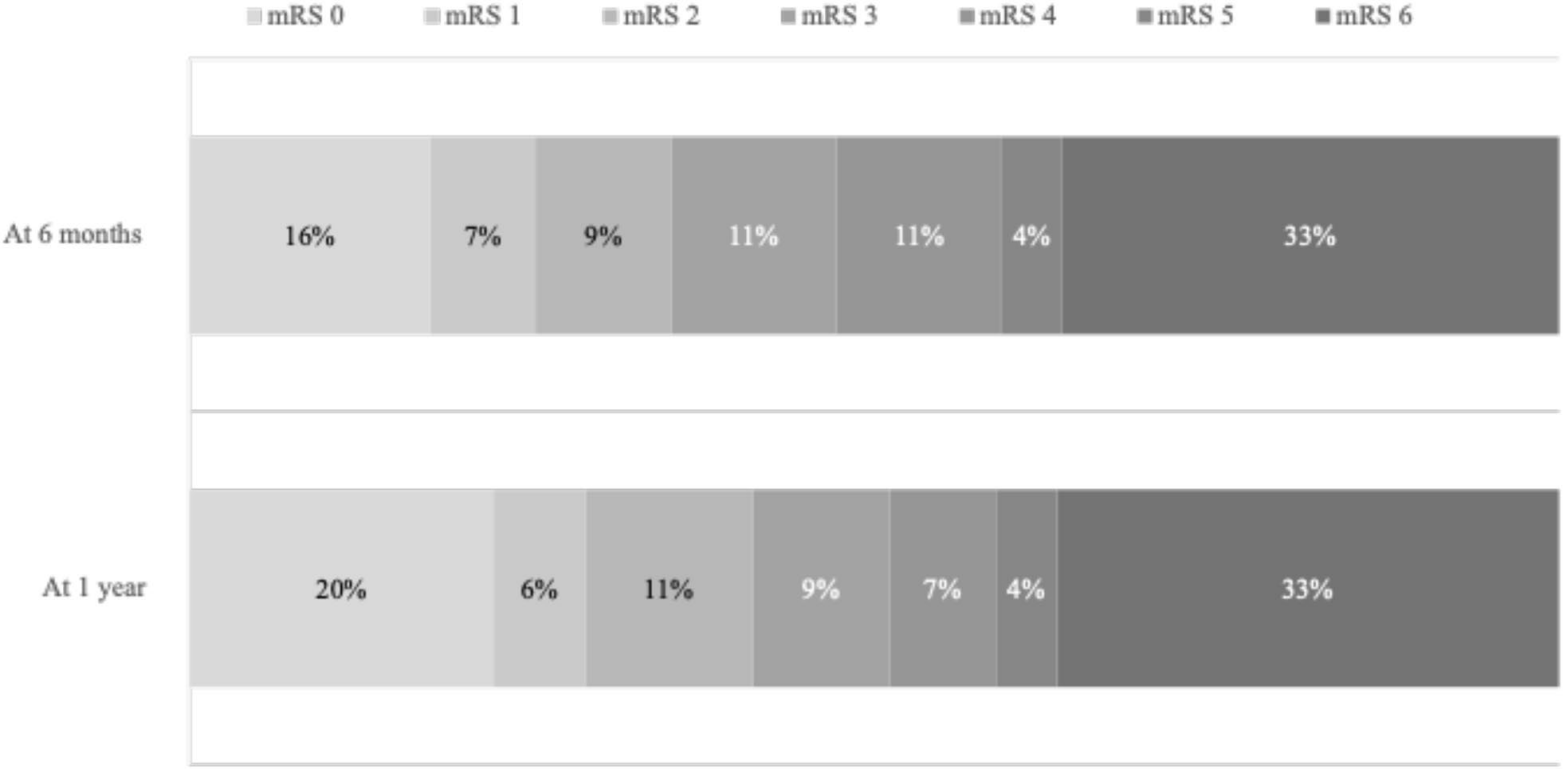
Neurologic outcome assessed with the modified Rankin Scale 6 months and 1 year after ICU discharge. Missing data: 5 (9%) patients at 6 months and 5 (9%) patients at 1 year. ICU: intensive care unit; VZV: varicella-zoster virus

ICU and hospital length of stay were 15 (6–38) days and 35 (17–55) days, respectively. Fourteen (25%) patients died during the ICU stay. Main causes of death were multi-organ failure in 4 (29%) patients (all of them had bacterial superinfection), treatment limitations because of an expected poor neurologic outcome in 4 (29) patients, brain death in 3 (21%) patients, and VZV-related fulminant hepatitis in 2 (14%) patients. Among the 41 (75%) ICU survivors, 4 (7%) with poor neurologic outcome ultimately died from sepsis during the first year after ICU discharge (lost to follow-up *n* = 5, 9%). Six months and 1 year after ICU discharge, 18 (33%) and 20 (36%) patients had a favorable neurologic outcome (mRS 0–2), while 14 (26%) and 12 (22%) had moderate-to-severe disability (mRS 3–5), respectively (Fig. 2, Additional file 1: Table S2).

### Factors associated with favorable one-year neurologic outcome

By univariate analysis, older age, underlying immunosuppression, and invasive mechanical ventilation were associated with an increased risk of disability or death 1 year after ICU discharge. By multivariable analysis, older age and invasive mechanical ventilation were independently associated with a lower probability of favorable neurologic outcome (mRS 0–2) (Table 3).

**Table 3:** Logistic regression analyses for factors associated with modified Rankin Scale scores of 0–2 at year-1 after ICU discharge

Variables	Univariate analysis		Multivariable analysis	
	OR [95% CI]	*p*-value	OR [95% CI]	*p*-value
Age, per year	0.93 [0.89–0.97]	0.0006	0.92 [0.88–0.97]	0.01
GCS at ICU admission, per point	1.13 [0.97–1.33]	0.12		
Focalization sign at ICU admission	1.93 [0.58–6.39]	0.27		
Seizures	1.46 [0.45–4.69]	0.52		
Status epilepticus	0.84 [0.21–3.36]	0.81		
SOFA at day 1, per point	0.88 [0.75–1.03]	0.1		
Maximal temperature at ICU admission, per degree	1.09 [0.65–1.82]	0.74		
Underlying immunosuppression	0.19 [0.05–0.79]	0.019	0.26 [0.04–1.59]	0.13
CSF elements, per element	1.00 [0.99–1.00]	0.40		
CSF protein level, per g/L	0.65 [0.39–1.09]	0.09		
Invasive mechanical ventilation	0.22 [0.05–1.04]	0.05	0.09 [0.01–0.84]	0.03
Renal replacement therapy	0.83 [0.23–2.99]	0.78		
Vasopressors	0.35 [0.10–1.22]	0.09		
Vasculitis on brain MRI	0.44 [0.076–2.576]	0.35		
Time from the onset of neurological symptoms to acyclovir, days	0.99 [0.91–1.08]	0.74		
High dose of steroids	0.71 [0.06–8.87]	0.78		

CSF, cerebrospinal fluid; GCS, Glasgow Coma Scale; ICU, intensive care unit; MRI, magnetic resonance imaging; SOFA, Sequential Organ Failure Assessment

## Discussion

### Key findings

In this multicenter retrospective cohort study, we used the ICD-10 coding system and a manual review of the medical charts to identify ICU patients with VZV encephalitis. We sought to describe their clinical features, management, and identify predictors of neurologic outcome 1 year after ICU discharge. We found that almost 80% of patients with VZV encephalitis had underlying immunosuppression. Moreover, we found that two-thirds of patients who underwent brain MRI had abnormal findings of whom 8 (15%) had signs of cerebral vasculitis. Finally, ICU mortality reached 25%, and a favorable neurologic outcome 1 year after ICU discharge was reported in 36% of the patients. Age and invasive mechanical ventilation were associated with an increased risk of disability or death.

### Relationship to previous studies

Acute viral encephalitis often requires ICU admission for monitoring and treatment [15]. Most of the current knowledge on VZV encephalitis relies on laboratory data with limited clinical information, studies conducted outside the ICU, or small case series [6–8, 16]. Thus, we report the largest cohort study on the clinical features and outcomes of patients admitted to the ICU for the management VZV encephalitis.

A recent study conducted in France on HSV encephalitis in the ICU setting found that patients had a median age of 64 years and 19% were immunocompromised [5]. In contrast, patients with VZV encephalitis in our study were younger (median age 53 years), and almost 80% were immunocompromised. Our results are in line with a recent nationwide Danish study where almost 40% of patients with VZV encephalitis had underlying immunosuppression [16]. VZV encephalitis results from reactivation of the virus located in neurons of dorsal root, autonomic and cranial ganglia, or less frequently from a primary infection [17]. Around 50% of herpes zoster cases occur in subjects with reduced cell-mediated immunity [18, 19]. Our results suggest that immunocompromised patients have an increased risk of developing more severe forms of VZV encephalitis [20]. Moreover, the high proportion of immunocompromised patients in our study could explain that most of them developed a disseminated form of the disease with typical chickenpox skin rash. Moreover, a recent study identified TLR3 mutations in patients with VZV and HSV encephalitis, suggesting a potential host factor for these diseases [21]. We cannot exclude that non-immunocompromised patients in our cohort had such risk factors for VZV encephalitis.

Neuroimaging is a key component to investigate acute encephalitis. In this setting, MRI is the best modality over CT to analyze the brainstem, deep brain structures, cerebellum, and document signs of vasculitis [7, 22–24]. In HSV encephalitis, brain MRI changes have been associated with 90 days poor prognosis [25]. In our study, 70% of the patients who underwent brain MRI had abnormal findings, of whom 50% had a normal CT. Moreover, we found that 8 (15%) had signs of cerebral vasculitis. Cerebral vasculitis is a well-known complication of VZV encephalitis with stenoses of the brain arteries often associated with ischemic lesions of the grey–white matter interface [22, 23]. A recent study found that 16% of the patients with VZV encephalitis had signs of vasculitis, and that vasculitis was independently associated with a higher risk of unfavorable outcome after discharge [16]. We did not find such association in our study. However, almost half of the patients included in our study did not have MRI investigations and thus, the true incidence of vasculitis in our cohort might be underestimated. We did not find any association between time from the onset of neurological symptoms to acyclovir treatment and neurological outcomes. However, most of the patients were treated within 72 h from the onset of symptoms. Moreover, we hypothesized that some symptoms were related to ischemic or inflammatory brain lesions, for which acyclovir treatment had no impact. These findings are in line with those reported in HSV encephalitis in the ICU setting [5].

The use of adjunctive treatment with steroids for VZV vasculitis remains a matter of debate. Although recommended by some authors [26–28], there are conflicting results and limited evidence to support the use of steroids in patients with herpes viruses encephalitis [29–31]. We did not find any association between the use of steroids and the neurologic outcome one year after discharge, yet the risk of unidentified confounders and selection bias precludes any conclusion. An ongoing randomized controlled trial testing dexamethasone compared with no intervention in patients with HSV encephalitis will provide further information on the impact of steroids on the neurologic outcome of patients with acute viral encephalitis (ClinicalTrials.gov number, NCT03084783).

Previous studies reported mortality rates ranging between 4 and 20% in patients with VZV encephalitis [16, 19, 20, 32]. In our study, we found a higher mortality rate of 25%. This figure might be explained by the fact that our study is the only one conducted in the ICU setting. Therefore, we may have selected a specific population with more severe forms of the disease. Jaquet et al*.* reported a mortality of 14% in ICU patients with HSV encephalitis [5]. Whether the higher rate of ICU mortality in our study is explained by specific characteristics of the VZV or by the clinical frailty associated with the immunocompromised status of most patients needs further investigation. Another important finding of our study is that only one-third of the patient had a favorable neurologic outcome 1 year after discharge. Furthermore, our longitudinal approach showed only a mild improvement of the neurologic outcome between 6 months and 1 year after ICU discharge (percentage of mRS 0–2 patients increased from 33 to 36%). This high rate of disability and death associated with VZV encephalitis is similar to the 71% of poor functional outcome reported by Jaquet et al*.* in ICU patients with HSV encephalitis. Herlin et al*.* reported that half of the 92 patients hospitalized for VZV encephalitis in Denmark had unfavorable outcome 3 months after discharge [16]. However, only 14% of these patients were admitted to the ICU, and outcome was categorized according to the Glasgow Outcome Scale, which makes comparisons difficult. We found age and invasive mechanical ventilation to be associated with poor neurologic outcome, which is in agreement with previous studies on acute viral encephalitis [4, 5, 16].

### Study implications

The findings of our study imply that VZV encephalitis in the ICU setting is predominantly diagnosed in immunocompromised patients who ultimately require invasive mechanical ventilation, and is associated with a high rate of disability and death. Moreover, they imply that brain MRI is a mandatory neuroimaging investigation for a thorough examination of the parenchyma and cerebral arteries because CT lacks sensitivity and accuracy to detect brain parenchyma inflammation, early signs of vasculitis, and cerebellum or brainstem lesions. Furthermore, our study implies that the diagnosis of cerebral vasculitis should be investigated systematically to discuss adjunctive immunomodulatory agents. Finally, the identification of age and invasive mechanical ventilation as predictors of functional outcomes may help physicians in their communication with patients and families about the likelihood of functional recovery.

## Strengths and limitations

Our study has several strengths. First, to our knowledge, we have conducted the largest study so far on the epidemiology and outcome of patients with VZV encephalitis admitted to the ICU. Thus, this study may help intensivists by providing new data relevant for their daily practice. Second, neurologic outcome was assessed twice, 6 months and 1 year after ICU discharge. Therefore, such longitudinal follow-up enabled us to detect patients who experienced late improvement. Third, we have a limited number of missing data for the assessment of neurologic outcome 1 year after ICU discharge (*n* = 5 patients). Fourth, we used a validated tool for the assessment of functional outcome, namely the modified Rankin Scale, which has demonstrated low inter- and intra-operator variability [33]. Finally, we identified predictors of neurologic outcome 1 year after ICU discharge which could help intensivists in their communication with patients and families about the prognosis of VZV encephalitis in the ICU setting.

This study also carries some limitations. First, the retrospective design implies information bias with a possibility of missing data. Second, brain MRI was performed in 55% of the patients. Thus, we may have underestimated the true incidence of some specific complications, such as VZV vasculitis. Third, we used the modified Rankin Scale to assess the neurologic outcome, which has limited sensitivity to identify mild-to-moderate cognitive sequelae. Therefore, we may have overestimated the percentage of patients who recovered without neurologic impairment. Fourth, we did not find any association between clinical parameters, laboratory data, EEG findings, treatments, and neurologic outcome. However, this lack of association might be related to the limited sample size of the study. Finally, we used a positive PCR for VZV in the CSF as inclusion criteria. Therefore, we might have missed some patients who could have been diagnosed with serologic testing of CSF.

## Conclusion

In conclusion, VZV encephalitis in ICU patients chiefly occurs in immunocompromised patients and is associated with a high rate of mortality and disability. Radiologic abnormalities of the brain parenchyma and vasculitis are common and should be investigated with MRI. One year after ICU discharge, 36% of the patients have a favorable neurologic outcome. Older age and invasive mechanical ventilation are associated with a higher risk of unfavorable neurologic outcome. Further research is needed targeting new therapeutic strategies to inhibit viral replication and reduce neuroinflammation, which hopefully could translate into better patients’ outcomes.

## Supplementary Information

**Additional file 1: Table S1.** Participating intensive care units. **Table S2.** Neurological outcome according to the treatment received.

## Data Availability

The datasets used and/or analyzed during the current study are available from the corresponding author on reasonable request.

## Abbreviations

AKI: Acute kidney injury
CNS: Central nervous system
CSF: Cerebrospinal fluid
CT: Computed-tomography
EEG: Electroencephalogram
GCS: Glasgow Coma Scale
HSV: Herpes simplex virus
ICD-10: International Classification of Diseases 10th Revision
ICU: Intensive care unit
IQR: Interquartile range
MRI: Magnetic resonance imaging
mRS: Modified Rankin Scale
OR: Odds ratios
PCR: Polymerase chain reaction
SOFA: Sequential Organ Failure Assessment
VZV: Varicella-zoster virus
