# Supplementary material for: Neurologic outcome of VZV encephalitis one year after ICU admission: a multicenter cohort study
Source: Ann Intensive Care. 2022 Apr 5;12:32. doi: 10.1186/s13613-022-01002-y (PMC8982685; doi:10.1186/s13613-022-01002-y)
Supplement: Supplementary file 1 — Additional file 1: Table S1. Participating intensive care units. Table S2. Neurological outcome according to the treatment received. [file 13613_2022_1002_MOESM1_ESM.docx]

**Additional file 1**

**Table S1: participating intensive care units**

| **Center** | **Patients (n)** |
| --- | --- |
| Hôpital Saint-Louis, Paris | 6 |
| Hôpital Saint-Antoine, Paris | 4 |
| Hôpital Bichat, Paris | 7 |
| Hôpital Cochin, Paris | 2 |
| Hôpital Pitié-Salpêtrière, Paris | 1 |
| CHU de Nantes, Nantes | 2 |
| CHU de Toulouse, Toulouse | 4 |
| CH de Versailles, Le Chesnay | 5 |
| Hôpital Henri Mondor, Créteil | 6 |
| Hôpital Herriot, Lyon | 4 |
| Hôpital de la Croix-Rousse, Lyon | 2 |
| Hôpital Nord, Marseille | 1 |
| CHU de Poitiers, Poitiers | 2 |
| CHU de Lille, Lille | 2 |
| CHU de Nancy, Nancy | 3 |
| CH d’Orléans, Orléans | 2 |
| CH de Mulhouse, Mulhouse | 1 |

**Table S2: Neurological outcome according to the treatment received.**

| **Variables** | **Number of patients** | | | **Patients with modified Rankin Scale scores of 0-2 at year-1, n%** | |
| --- | --- | --- | --- | --- | --- |
| **Treatment**, n (%) |  | | |  | |
| Acyclovir monotherapy | | 42 | 17 (47.6%) | |  |
| Acyclovir and foscavir | | 6 | 1 (16.6%) | |  |
| Acyclovir and ganciclovir | | 2 | 1 (50%) | |  |
| Acyclovir and specific intravenous immunoglobulins | | 2 | 0 (0%) | |  |
| Acyclovir and high dose of steroids | | 3 | 1 (33.3%) | |  |
| Acyclovir and specific intravenous immunoglobulins and high dose of steroids | 1 | | | 0 (0%) | |
